# Reduced contrast sensitivity, pattern electroretinogram ratio, and diminished a-wave amplitude in patients with major depressive disorder

**DOI:** 10.1007/s00406-024-01826-8

**Published:** 2024-05-28

**Authors:** Evelyn B. N. Friedel, Ludger Tebartz van Elst, Malina Beringer, Dominique Endres, Kimon Runge, Simon Maier, Jürgen Kornmeier, Michael Bach, Katharina Domschke, Sven P. Heinrich, Kathrin Nickel

**Affiliations:** 1 Department of Psychiatry and Psychotherapy, Medical Center - University of Freiburg, Faculty of Medicine, University of Freiburg, Freiburg, Germany; 2 Eye Center, Medical Center - University of Freiburg, Faculty of Medicine, University of Freiburg, Freiburg, Germany; 3 Faculty of Biology, University of Freiburg, Freiburg, Germany; 4 German Center for Mental Health (DZPG), Partner Site Berlin, Berlin, Germany; 5 Institute for Frontier Areas of Psychology and Mental Health, Freiburg, Germany

**Keywords:** PERG, Electroretinogram, Depression, A-wave, Photopic negative response, Contrast sensitivity

## Abstract

The electroretinogram (ERG), a non-invasive electrophysiological tool used in ophthalmology, is increasingly applied to investigate neural correlates of depression. The present study aimed to reconsider previous findings in major depressive disorder (MDD) reporting (1) a diminished contrast sensitivity and (2) a reduced patten ERG (PERG) amplitude ratio, and additionally, to assess (3) the photopic negative response (PhNR) from the flash ERG (fERG), with the RETeval® device, a more practical option for clinical routine use. We examined 30 patients with a MDD and 42 healthy controls (HC), assessing individual contrast sensitivity thresholds with an optotype-based contrast test. Moreover, we compared the PERG ratio, an established method for early glaucoma detection, between both groups. The handheld ERG device was used to measure amplitudes and peak times of the fERG components including a-wave, b-wave and PhNR in both MDD patients and HCs. MDD patients exhibited diminished contrast sensitivity together with a reduced PERG ratio, compared to HC. With the handheld ERG device, we found reduced a-wave amplitudes in MDD, whereas no significant differences were observed in the fERG b-wave or PhNR between patients and controls. The reduced contrast sensitivity and PERG ratio in MDD patients supports the hypothesis that depression is associated with altered visual processing. The findings underscore the PERG’s potential as a possible objective marker for depression. The reduced a-wave amplitude recorded with the RETeval® system in MDD patients might open new avenues for using handheld ERG devices as simplified approaches for advancing depression research compared to the PERG.

**Supplementary Information:**

The online version contains supplementary material available at 10.1007/s00406-024-01826-8.

## Introduction

Major depressive disorder (MDD) is a widespread mental health condition with a significant disease burden and a global point prevalence of about 4.7% [1, 2]. The diagnosis of MDD primarily relies on clinical assessment, as there is currently no established biomarker or combination of biomarkers for routine clinical diagnostic use or therapy monitoring [3]. MDD has been associated with specific alterations in visual functions, such as changes in the cortical processing of visual stimuli [4], contrast discrimination [5], and features of the electroretinogram (ERG) [6], highlighting the disorder's impact on visual signal transduction.

In early studies analyzing visual contrast processing, elevated contrast discrimination thresholds could be detected in medicated and unmedicated MDD patients compared to healthy controls (HC) [5]. This finding closely resembles earlier observations in patients with Parkinson's disease [7], supporting the hypothesis of a possible altered dopaminergic neurotransmission in depression. Fam et al. [8] confirmed the finding of reduced visual contrast sensitivity in MDD patients performing the optotype-based Landolt-C contrast test from the Freiburg Visual Acuity and Contrast Test (FrACT) [9].

The ERG is a non-invasive ophthalmological examination method of the retina, which is increasingly applied in the field of neuropsychiatric research [10–13]. Given its embryological origin as part of the central nervous system [14], the retina is often regarded as an easily accessible “window to the brain” [15]. Studies suggest that changes in dopaminergic neurotransmission in the brain, which might be associated with the etiology of mental disorders such as depression, can also affect retinal processing and thus affect ERG responses [16].

Applying the pattern ERG (PERG), retinal ganglion cells are stimulated by local contrast changes in alternating black/white checkerboard reversal stimuli, whereby the average luminance across the alternating checkerboard stimuli stays constant [17]. Higher stimulation frequencies (> 6 reversals per second [rps]) lead to a sinusoidal PERG response, the steady-state PERG (ssPERG) [18]. The ganglion cell response to the local contrast changes is then reflected in the magnitude of the PERG response at the checkerboard stimulation frequency.

The components of the flash ERG (fERG) can be used to measure the signal processing on different retinal levels. In the fERG of a light adapted retina (LA-fERG), for instance, the a-wave reflects the activity of the cone photoreceptors and Off-bipolar cells, the b-wave of the On- and Off-bipolar cells and the photopic negative response (PhNR) corresponds to the activity of the retinal ganglion cells [19, 20]. Despite the PhNR of the fERG and the PERG reponse are both associated with retinal ganglion cell function, the fERGs are generated from the entire retina with minimal macula contribution [20] while the large-field PERG is based on central and paracentral macular function [21].

Previous studies showed a reduced slope of the ssPERG contrast gain function in both medicated and unmedicated patients with MDD compared to HC, which correlated with the severity of depressive symptoms [22]. In a follow-up study, a normalization to HC level was found with remission of depression [23]. This finding renders the PERG a promising tool for identifying potential biomarkers for depression. However, Fam and colleagues [8] reported normal ssPERG contrast gain in their MDD group.

PERG responses are not only modulated by checkerboard contrast, but also by check size and stimulation frequency. In a previous study, we explored different stimulation settings for ssPERG recordings [24]. We observed that the most prominent difference between MDD and HC occurs at a slightly higher stimulation frequency (18.75 rps) than previously used (12.5 rps in Bubl et al. [22]). Calculating an amplitude ratio of ssPERG responses from a finer compared to a coarse (only four checks on screen) checkerboard pattern (ssPERG ratio: check size 0.8°/16°) further improves the distinction between groups, most likely due to a reduction in inter-individual variability [24]. These parameter settings for PERG recordings are strongly reminiscent of the “PERG ratio” protocol, applied for early glaucoma detection [25–27].

Other studies used the fERG alone or alongside PERG recordings in MDD patients [6, 28–30]. Unlike the above introduced PERG paradigms, which typically use checkerboard stimuli, the conventional ERG is recorded in response to flash stimuli. Flash ERG is advantageous for assessing the integrity of retinal signals at different retinal levels, because proper fixation during recording and participant’s visual acuity are less critical. Moreover, handheld mydriasis-free devices, like the RETeval® system (LKC Technologies, Gaithersburg, MD, USA), may be a promising flash-ERG-tool, suitable for routine clinical use [31].

In the largest study analyzing 100 MDD patients and 100 HC with the light- and dark-adapted fERG (LA- and DA-fERG), Hébert and colleagues [30] reported increased b-wave peak times in medicated patients (N = 83) in LA-fERG and reduced DA-fERG a-wave amplitudes in both medicated and unmedicated (N = 17) MDD patients, compared to the HC group. Reduced amplitudes of the a- and b-waves of the LA-fERG (reflecting cone responses) were found only in unmedicated MDD patients, suggesting possible normalization with medication [30]. Cosker and colleagues [28] reported shorter a- and b-wave peak times in MDD for both the DA- and LA-fERG using the standard 3 cd∙s∙m^−2^ flash strength (DA3- LA3-ERG). In both conditions the b-wave amplitude was enhanced in MDD, whilst a larger a-wave amplitude was observed only in the LA3-fERG [28]. Using the RETeval® system for fERG recordings with white and additional red flashes (particularly suitable for ganglion cell stimulation [19]), Demmin and colleagues [29] found a reduced implicit time of the PhNR, reflecting ganglion cell activity, in MDD, while all other fERG components in MDD patients closely resembled HC responses [29].

### Aims of the Study

The present study has the following aims: Replicating previous findings of (1) a reduced contrast sensitivity [5, 8] and (2) ganglion cell response [22, 24] in MDD patients compared to HC by evaluating the ssPERG ratio in a larger independent sample. (3) Exploring the feasibility of the handheld RETeval® system [32] for fERG recordings via the PhNR as a correlate of ganglion cell function. Advantages of the RETeval® system are a reduction of measurement time by a third compared to the complex classical PERG setup. This is crucial for patients with mental disorders and limited attention span. Moreover, precise fixation, proper refraction and extensive patient cooperation are less critical for signal quality. Given that RETeval® measurements are conventionally conducted using skin electrodes, the procedure offers enhanced comfort relative to the traditional corneal contact DTL electrodes for PERG assessment. If comparably consistent findings (contrast between MDD patients and HCs) are achieved with the RETeval® system and the classical ssPERG paradigm, the RETeval® system could significantly enhance the integration of retinal assessments into psychiatric routine clinical practice.

## Materials and methods

### Participants

The study was conducted after approval by the Ethics Committee of the University of Freiburg (Approval ID: 314/18). All patients gave written informed consent to participate. The study was performed in accordance with the Declaration of Helsinki [33].

The examinations were conducted at the Department of Psychiatry and Psychotherapy of the University Medical Center Freiburg, Germany. Patients who met diagnostic criteria according to the International Classification of Diseases, 10th revision (ICD-10) for a severe depressive episode (ICD-10: F32.2) or a recurrent depressive disorder, with a current severe episode without psychotic symptoms (ICD-10: F33.2) were included. Diagnosis was clinically established by an experienced senior psychiatrist. The Montgomery-Åsberg Depression Rating Scale (MADRS) [34] was employed as an external assessment scale by an experienced senior psychiatrist. The Beck Depression Inventory (BDI-II) [35, 36] was used to assess self-reported severity of depressive symptoms. To rule out comorbid autism spectrum disorder, the Autism-Spectrum Quotient (AQ) [37] and the Empathy Quotient (EQ) [38] were assessed. The presence of attention-deficit/hyperactivity disorder (ADHD) in childhood was excluded with the Wender Utah Rating Scale (WURS-k) [39]. The Structured Clinical Interview for DSM (SCID-I and -II; [40]) and the Symptom Checklist (SCL-90-R; [41]) were additionally collected to screen participants for general psychiatric diseases.

MDD patients were allowed to take a medication with a selective serotonin reuptake inhibitor (SSRI), a serotonin and norepinephrine reuptake inhibitor (SNRI), or mirtazapine for a maximum of 14 days.

The HC group was matched to the patient group based on age and sex. The presence of any psychiatric disorder was defined as exclusion criterion for HCs. The HC group completed the same self-reporting questionnaires as the patients with depression.

All participants were aged > 18 years. Exclusion criteria for all study participants included the presence of psychotic symptoms, bipolar disorder, somatic diseases such as arterial hypertension or diabetes mellitus as well as seizures or substance abuse. Further, any ophthalmologic diseases including glaucoma (excluding correctable refraction errors), myopia exceeding − 6 dpt, or hyperopia exceeding + 6 dpt, or a decimal visual acuity < 0.8 were exclusion criteria for both groups. Participants additionally received optical coherence tomography examinations (not analyzed here), which were screened by an ophthalmologist. fERG and PERG data from eyes showing ophthalmological findings requiring further specialist clarification were excluded from the analysis.

## Psychophysics

### Visual acuity and contrast sensitivity

Visual acuity and contrast sensitivity thresholds were assessed with the FrACT [9], a computer-based semi-automatic visual test battery including different optotypes or pattern stimuli. We used Landolt-C optotypes in eight orientations for both tests presented on a monitor (31 × 17.5 cm, 1920 × 1080 pixels) at 180 cm distance. Following a forced-choice paradigm, participants were instructed to press the corresponding button on a numpad according to the positions of the Landolt-Cs’ gap [42]. The presentation sequence is based on an adaptive staircase methodology, the Best-Pest (best parameter estimation by sequential testing) algorithm [9]. We used 18 trials (optotype presentations) in both tests. Visual acuity was evaluated monocularly using refraction correction if necessary to achieve a minimum decimal visual acuity of 0.8 for each eye. Weber contrast thresholds were tested binocularly using a constant large (50 arcmin diameter) Landolt-C optotype. The Landolt-C contrast test of the FrACT yields results close to chart contrast tests like the Mars chart or Pelli-Robson chart [43].

### PERG

PERG recording was conducted following the recommendations of the International Society for Clinical Electrophysiology of Vision (ISCEV) [18, 44] using the EP2000 acquisition module [45] for stimulation and recording. Corneal DTL electrodes [46] were positioned along the lower eyelid with contact to the cornea for recording. Reference electrodes were placed at the ipsilateral canthi, an ear clip electrode was used as the ground. Stimuli were presented at 57 cm distance on a Cathode Ray Tube monitor (75 Hz refresh rate; 800 × 600 pixels) covering a field size of 38 × 29° (4:3 aspect ratio) corresponding to a large field PERG stimulation. Amplified (50-fold) signals were digitized at 1 kHz and 16-bit resolution.

#### Steady-state stimulation

Two black/white alternating checkerboard patterns with different check sizes were used for stimulation and to calculate the ssPERG ratio. We presented a fine pattern (check size 0.8°) and a coarse pattern (check size 19° ‒ only four checks on screen), both with a Michelson contrast of about ≈ 100% and a reversal rate of 15/s (15 rps) according to a steady-state stimulation. Both check sizes (0.8° and 19°) were presented in interleaved blocks (5.3 s duration, consisting of 5 consecutive sweeps with a duration of 1065 ms each) to collect a minimum of 80 artifact-free trials for stimulus-synchronized averaging. Artifact contaminated sweeps were automatically rejected during recording (± 120 µV threshold). Participants were instructed to fixate a centrally displayed cross during recording and to report the digits that randomly appeared within the fixation cross.

#### Data extraction

The “EP2000” analysis module [45] within IGOR Pro 7 by Wave Metrics® was used for first offline data processing and inspection of individual recordings. A discrete Fourier transformation was conducted following the removal of any linear trends, resulting, e.g., from baseline drifts [47]. Steady-state PERG amplitudes were extracted from the Fourier spectra at 15 Hz (corresponding to 15 rps) and noise corrected [47].

### Flash ERG with RETeval®

LA-fERG was recorded with the handheld RETeval® device from LKC Technologies (firmware version 2.13.1) [32] using a Troland-based stimulation protocol, where flash strengths automatically compensate for pupil size, eliminating the need for mydriasis. A red LED within the RETeval® served as fixation target. Sensor-strip skin electrodes placed about 2 mm under the lower eyelid were used for signal recording. After 10 min of light adaptation (normal room lighting conditions (500 lx)), the fERG measurement was conducted according to the recommendations of the ISCEV [20].

#### PhNR stimulation

Following the standard procedure described by the ISCEV for a particularly suitable PhNR stimulation [19], 200 red flashes (621 nm; 38 Td·s) were presented on a blue background light (470 nm; 380 Td) with a stimulation frequency of 3.43 Hz.

#### Data extraction

Prior to data export, each recording was individually checked for artifacts, baseline drifts, and accurate detection of the fERG components (a-wave, b-wave and the PhNR). Flash ERG data were extracted using the RETeval® RFF Extractor® software (version 2.13.0.0) provided by LKC Technologies Inc.. Peak amplitudes in microvolts (µV) and corresponding peak times in milliseconds (ms) were extracted for the a-wave, b-wave, and the PhNR. The a-wave amplitude was defined as the first negative trough relative to the baseline. The b-wave amplitude is the subsequent maximum positive deflection calculated from the a-wave minimum, followed by the PhNR, which was measured in relation to baseline in two different ways: (1) PhNR at minimum (min.), according to the negative trough between 55 and 100 ms, or (2) PhNR at 72 ms [48].

### Statistical analysis

Statistical analysis and graphical representations were conducted with “R” in RStudio [49] using the “tidyverse” [50] core packages.

#### Psychophysics – contrast sensitivity

Individual Weber contrast thresholds (C_Weber_) from the Landolt-C contrast test using the FrACT [9, 43] were converted to logarithmic Weber contrast sensitivity (logCS_Weber_ = log_10_(1/C_Weber_)) as described by Bach et al. [51].

#### Electrophysiological examinations

Contaminated ssPERG and fERG recordings (e.g. baseline-drifts, elevated artifacts etc.) from individual eyes were excluded from further analysis. If available, data of both eyes were averaged for all participants.

*Steady-state PERG* amplitudes, were summarized as the ssPERG ratio according to the ratio between amplitudes in response to the finer compared to the coarser pattern (ssPERG ratio: 0.8°/19°), following the “PERG ratio protocol” for early glaucoma detection [25, 26] and our previous investigation in patients with MDD [24].

*Flash ERG (RETeval®)* amplitudes from the different PhNR measures (at min. or at 72 ms) were also used to calculate ratios. The “W-ratio” ((b-wave amplitude – PhNR at min.)/(b-wave – a-wave amplitude)) was computed for the PhNR at min. following the recommendations of Mortlock et al. [52]. The “P-ratio” (PhNR at 72 ms/b-wave amplitude) was calculated for the PhNR at 72 ms as described in Preiser et al. [48]. As secondary outcomes, we subsequently analyzed peak amplitudes of the a- and b-wave as well as the peak times of the a- and b-wave and the PhNR at minimum.

Statistical comparisons between MDD and HC were based on differences in medians (MDD − HC) and were carried out using the “infer” package [53] using permutation tests (10,000 replicates) to calculate *p*-values. Based on previous literature one-sided *p*-values were computed for the contrast sensitivity (logCS_Weber_) and the ssPERG ratios assuming a reduction in both measures in the MDD group. No assumptions were made for the other comparisons.

Significance level was defined as α = 0.05 and adjusted for the false discovery rate (FDR) [54] taking the three primary outcome variables into account. No FDR adjustments were made for the additional fERG parameters (secondary outcomes) such as the a- and b-wave amplitudes or peak time measures.

For both groups two-sided median-based 95% confidence intervals (95% CI) were computed using a bootstrapping procedure (10,000 replicates). To estimate the magnitude of MDD alterations compared to HC, the proportional deviation of the MDD medians from the HC medians was calculated in % for the PERG and fERG data (“MDD vs. HC”). For the logarithmic contrast sensitivity, the absolute difference between group medians was determined.

Spearman’s correlation coefficients *rho* were computed with the “correlation” package [55] using the “jumOutliere” package [56] to calculate *p*-values based on permutations (10,000 replicates).

For correlation analysis, we considered the three primary endpoints (contrast sensitivity, ssPERG ratio, PhNR measures) together with the severity of depressive symptoms, evaluated with the BDI-II for all participants, or using the MADRS scores, which were available for MDD only.

#### Antidepressant medication

We additionally report descriptive statistics for the medicated and unmedicated subsamples of MDD patients (Supplementary Fig. 1, Supplementary Table 1).

## Results

### Participants

Forty-two patients with MDD were initially recruited for study participation. Subsequently, six patients declined to participate, one aborted the examination, three were excluded due to their psychiatric medication (bupropion, opipramol, trimipramine), one because of low visual acuity in both eyes (0.6 and 0.4) and another due to ophthalmological findings in both eyes (suspicion of domed-shaped macula).

Finally, 30 patients with MDD (21 female) and 42 HC (29 female) were included in the study. Of the MDD patients, 16 were diagnosed with a severe depressive episode (ICD-10: F32.2), 14 with a recurrent depressive disorder, current episode severe without psychotic symptoms (ICD-10: F33.2). Nine patients were naïve to psychiatric medication while 21 took antidepressants, with none starting medication more than 14 days prior to the study. The medication regiments included SSRIs alone (N = 2; 10%), SSRIs in combination with mirtazapine (N = 4; 19%), SNRIs alone (N = 4; 19%) or in combination with mirtazapine (N = 5; 24%), and mirtazapine alone (N = 6; 29%). MDD and HC groups were matched according to sex and age. The demographic and psychometric data are summarized in Table 1.

**Table 1:** Demographic and psychometric data

Parameter	MDD (N = 30)	HC (N = 42)	*p*-value (sig. level)
Sex: female Sex: male	21/30 (70%) 9/30 (30%)	29/42 (69%) 13/42 (31%)	.421 (ns)
Age in years	32 (22, 39); 19 − 65	30 (23, 35);19 − 65	.678 (ns)
ICD-10 Diagnosis: F32.2 ICD-10 Diagnosis: F33.2	16 (53%) 14 (47%)	−	−
With antidepressant medication Naïve to antidepressant medication	21/30 (70%) 9/30 (30%)	−	−
Medication duration [days]	7 (4, 11); 1 − 14	−	−
MADRS	36 (35, 40)^+^; 28 − 45	−	−
BDI-II	26 (22, 38)^+^; 14 − 53	2 (0, 4); 0 − 13	< .001 (*)
AQ	20 (16, 22)^+^; 9 − 28	22 (18, 24); 9 − 27	.262 (ns)
EQ	44 (38, 49)^+^; 27 − 53	46 (43, 48); 30 − 59	.315 (ns)
WURS-k	11 (4, 26)^+^; 0 − 53	8 (4, 14); 0 − 30	.436 (ns)

Numerical data are summarized as median (1st and 3rd quartiles) and range, categorical data as number of counts and proportion in %. *P*-values were calculated using permutations (10,000 replicates), employing Chi-Squared tests for categorical data and differences in medians (MDD − HC) for numerical data.

*AQ* Autism-Spectrum Quotient; *BDI-II* Beck Depression Inventory II; *EQ* Empathy Quotient; *F32.2* severe depressive episode without psychotic symptoms (ICD-10); *F33.2* recurrent depressive disorder, current episode severe without psychotic symptoms (ICD-10); *ICD-10* International Statistical Classification of Diseases and Related Health Problems version 10; *MADRS* Montgomery-Åsberg Depression Rating Scale; *N* number of observations; *ns* not significant; *WURS-k* Wender Utah Rating Scale. ^+^ 1 missing data set; * statistically significant difference

### Contrast sensitivity

**Fig. 1:**
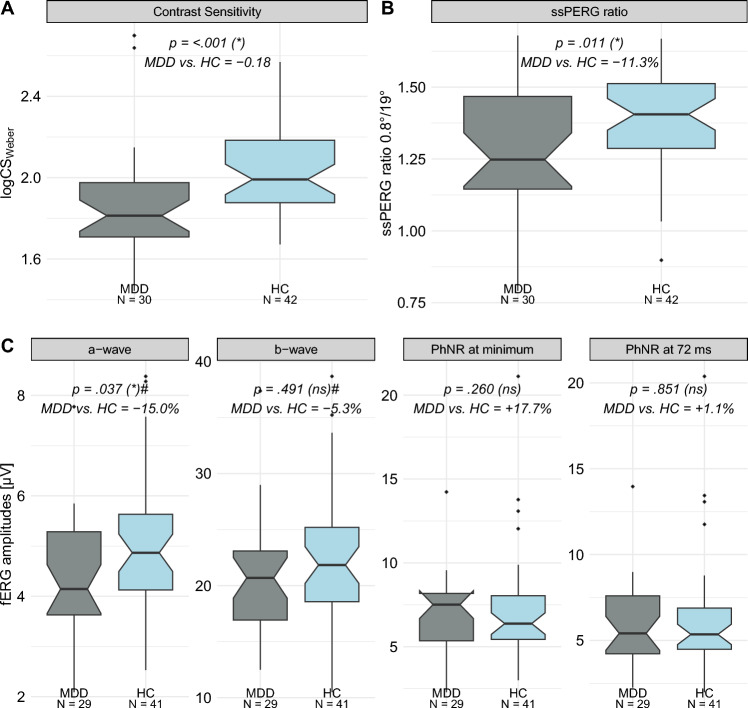
Psychophysical and electrophysiological results comparing MDD and HC. **A** Logarithmic Weber contrast sensitivity (logCS_Weber_); **B** ssPERG ratio; **C** Peak amplitudes in µV of the fERG components: a-wave, b-wave, photopic negative response (PhNR) at minimum (min.) and at 72 ms. *P*-values were calculated by permutations (10,000 replicates) for the differences in medians (MDD − HC). Significance levels in brackets were FDR-adjusted considering 3 primary outcomes (logCS_Weber_, ssPERG ratio and the PhNR) and not adjusted for secondary outcome variables (#). MDD vs. HC describes the difference in the logCS_Weber_ scores between MDD and HC or the relative deviations of the MDD’s from the HC’s medians in % for the other data. *fERG* flash Electroretinogram; *FDR* false discovery rate; *HC* healthy controls; *logCS*_*Weber*_ logarithmic Weber contrast sensitivity; *MDD* patients with major depressive disorder; *N* number of observations; *ns* not significant; *PhNR* photopic negative response; *ssPERG* steady-state pattern Electroretinogram; * statistically significant difference; # not adjusted

The psychophysical contrast test (FrACT; [9]) revealed a significantly reduced contrast sensitivity (logCS_Weber_) in patients with MDD (N = 30) compared to HC (N = 42) (MDD−HC = ‒ 0.18; *p* < 0.001) (Fig. 1A, Table 2).

**Table 2:** Results of the psychophysical and electrophysiological examinations comparing MDD and HC

Test & Parameter		MDD median [95% CI] (N = 30)	HC median [95% CI] (N = 42)	MDD vs. HC	*p*-value (sig. level)
Psychophysics: contrast sensitivity [logCS_Weber_]^1^		1.81 [1.75, 1.95]	1.99 [1.92, 2.04]	− 0.18	< 0.001 (*)
ssPERG	Check size				
ssPERG ratio^1^	0.8°/19°	1.25 [1.16, 1.41]	1.41 [1.35, 1.44]	− 11.3%	0.011 (*)
Amplitude [µV]	0.8°	3.38 [3.00, 3.84]	3.68 [3.30, 4.19]	− 8.2%	0.237 (ns)^#^
	19°	2.65 [2.45, 2.95]	2.72 [2.41, 2.85]	− 2.6%	0.408 (ns)^#^
RETeval®	fERG peak	MDD (N = 29)	HC (N = 41)		
PhNR amplitude [µV]	PhNR at min	7.51 [5.58, 8.03]	6.38 [5.64, 7.20]	+ 17.7%	0.260 (ns)
	PhNR at 72 ms	5.41 [4.38, 6.37]	5.35 [4.77, 5.75]	+ 1.1%	0.851 (ns)
PhNR ratios	W-ratio	1.12 [1.09, 1.16]	1.07 [1.04, 1.09]	+ 4.7%	0.047 (ns)
	P-ratio	0.26 [0.20, 0.33]	0.25 [0.22, 0.27]	+ 4.0%	0.468 (ns)
Amplitudes [µV]	a-wave	4.14 [3.85, 4.62]	4.87 [4.46, 5.24]	− 15.0%	0.037 (*)^#^
	b-wave	20.69 [19.06, 21.99]	21.84 [19.41, 23.62]	− 5.3%	0.491 (ns)^#^
Peak time [ms]	a-wave	11.40 [11.07, 11.77]	11.72 [11.57, 12.00]	− 2.7%	0.056 (ns)^#^
	b-wave	27.25 [27.06, 27.67]	27.34 [26.99, 27.51]	− 0.3%	0.725 (ns)^#^
	PhNR at min	71.11 [64.39, 72.60]	68.58 [64.91, 71.48]	+ 4.2%	0.445 (ns)^#^

Median and bootstrapped (10,000 replicates) 95% confidence intervals (CIs) for both groups are depicted. The difference of the logCS_Weber_ and the relative deviation of the MDD’s compared to HC’s medians in % for electrophysiological data are shown (MDD vs. HC). *P*-values were calculated by permutations (10,000 replicates) for differences in medians (MDD − HC). Significance levels in brackets were FDR-adjusted for the primary outcome variables (logCS_Weber_, ssPERG ratio and PhNR amplitude) and not adjusted (^#^) for secondary parameters.

^1^ one-sided tests assuming lower MDD medians; *fERG* flash electroretinogram; *FDR* false discovery rate; *HC* healthy controls; *logCS*_*Weber*_ logarithmic Weber contrast sensitivity; *MDD* patients with major depressive disorder; *N* number of observations; *ns* not significant; *P-ratio* PhNR amplitude at 72 ms/b-wave amplitude; *PhNR* photopic negative response; *ssPERG* steady-state pattern electroretinogram; *W-ratio* (b-wave amplitude − PhNR amplitude at min.)/(b-wave amplitude − a-wave amplitude); * statistically significant difference; ^#^ not adjusted

### Electrophysiological evaluations (ssPERG and fERG)

Prior to averaging data from both eyes, we had to exclude data of individual eyes. One HC had an ophthalmological finding (small pigment epithelial alteration with associated photoreceptor atrophy in the papillomacular bundle) in one eye. In the MDD group, one patient had uncorrectable low visual acuity in one eye (0.6), another patient had an ophthalmological finding in one eye (sub- and perifoveal pigment epithelial alterations).

### ssPERG ratio (0.8°/19°)

Data from four HCs and two patients showed excessive baseline drifts in one eye and were rejected from further ssPERG analysis, resulting in 79 eyes from HC and 56 eyes from patients with MDD that could be considered for ssPERG analysis.

Comparing the ssPERG ratio (0.8°/19°) between both groups revealed a significant reduction in the ssPERG ratio in patients with MDD (N = 30) compared to HCs (N = 42) (MDD vs. HC: − 11.3%; *p* = 0.011) (Fig. 1B, Table 2).

Although the primary focus was on the ssPERG ratio, the raw ssPERG amplitudes for individual check sizes were also evaluated. No significant differences were found in raw ssPERG amplitudes between MDD patients and HC for either the fine 0.8° (*p* = 0.237) or the coarse 19° check size (*p* = 0.408). However, it is noteworthy, that the reduction in the raw ssPERG amplitudes in MDD patients were more pronounced for the finer compared to the coarser pattern (MDD vs. HC: 0.8°: − 8.2%; 19°: − 2.6%; Table 2).

### Flash ERG (RETeval®)

In the HC and the MDD group, one participant had to be excluded due to artifact-contaminated recordings in both eyes that prevented proper peak detection. Additionally, two single eyes of HCs and five single eyes of MDD patients were excluded for the same reasons. Thus, data from 29 MDD (51 eyes) patients and 41 HC (79 eyes) were included in the fERG analysis.

#### Flash ERG amplitudes

Regarding our primary endpoint, the PhNR, a correlate of ganglion cell function, no differences were found between MDD patients and HCs, neither in the PhNR at min. (MDD vs. HC: + 17.7%; *p* = 0.260), nor measured at 72 ms (MDD vs. HC: + 1.1%; *p* = 0.851) (Fig. 1C, Table 2). Similarly, after FDR-adjustment, no significant group differences were found in the PhNR ratios comparing MDD and HC (MDD vs. HC: W-ratio: + 4.7%; *p* = 0.047; P-ratio: + 4.0%; *p* = 0.468) (Table 2).

For the secondary outcomes, the a- and b-wave amplitudes, a significant reduction of the a-wave amplitude was observed in the MDD group compared to the HC group (MDD vs. HC: − 15.0%; *p* = 0.037). The b-wave amplitude between MDD and HC group was not significantly different (MDD vs. HC: − 5.7%; *p* = 0.491) (Fig. 1C, Table 2).

#### Flash ERG peak times

The fERG peak times, another secondary outcome, were not significantly different between patients with MDD and HCs. Neither the a-wave (MDD vs. HC: − 2.7%; *p* = 0.056), or b-wave (MDD vs. HC: − 0.3%; *p* = 0.725), nor the PhNR at minimum (MDD vs. HC: + 4.2%; *p* = 0.445) revealed any differences in peak time between groups (Table 2).

### Spearman correlation coefficients

**Fig. 2:**
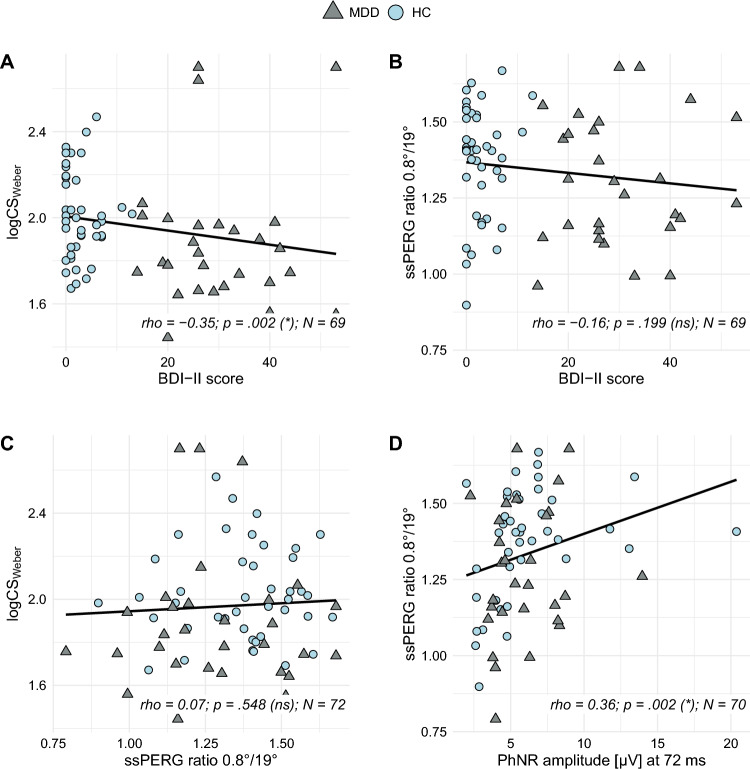
Selection of scatterplots illustrating relationships between tested parameters. Spearman’s *rho* was computed for correlation analysis. *P*-values were calculated by permutations (10,000 replicates). FDR-adjusted significance levels in brackets. The original data is shown here, not the rank-transformed data. **A** Contrast sensitivity (logCS_Weber_) and severity of depressive symptoms (BDI-II scores); **B** ssPERG ratio and severity of depressive symptoms (BDI-II scores); **C** Contrast sensitivity (logCS_Weber_) and the ssPERG ratio; **D** Both retinal ganglion cell responses are depicted, the ssPERG ratio and the PhNR amplitude at 72 ms. *BDI-II* Beck Depression Inventory II; *logCS*_*Weber*_ logarithmic Weber contrast sensitivity; *N* number of observations; *ns* not significant; *PhNR* photopic negative response; *ssPERG* steady-state pattern electroretinogram; * statistically significant difference

Considering all participants, MDD and HC, we analyzed relations between the severity of depressive symptoms, according to the BDI-II scores, and the contrast sensitivity (logCS_Weber_) or the ssPERG ratio (both reduced in MDD). A significant correlation was found between the BDI-II scores and the contrast sensitivity (*rho* =  − 0.35; *p* = 0.002; Fig. 2A and Table 3), but no correlation was observed between the severity of depressive symptoms and the ssPERG ratio (*rho* =  − 0.16; *p* = 0.199; Fig. 2B and Table 3).

**Table 3:** Spearman's correlation coefficients (*rho*)

Pairs of correlation analysis		Spearman’s *rho*	*p*-value (sig. level)	N
Test variable 1	Test variable 2			
logCS_Weber_	BDI-II	− 0.35	0.002 (*)	69
	MADRS	‒0.35	0.074 (ns)	28
	ssPERG ratio	0.07	0.548 (ns)	72
ssPERG ratio	BDI-II	− 0.16	0.199 (ns)	69
	MADRS	0.14	0.462 (ns)	28
	PhNR at min	0.27	0.023 (ns)	70
	PhNR at 72 ms	0.36	0.002 (*)	70
	W-ratio	0.11	0.345 (ns)	70
	P-ratio	0.23	0.051 (ns)	70
a-wave amplitude (fERG)	BDI-II	− 0.23	0.063 (ns)^#^	67
	MADRS	− 0.14	0.494 (ns)^#^	27
	logCS_Weber_	0.08	0.533 (ns)^#^	70
	ssPERG ratio	0.07	0.589 (ns)^#^	70

Contrast sensitivity (logCS_Weber_) and the ssPERG ratio were analyzed for correlations with the severity of depressive symptoms (BDI-II and MADRS scores), and for correlation between each other. The different measures of the ganglion cell responses were additionally tested comparing the ssPERG ratio with the different PhNR measures. A-wave amplitudes were investigated for correlations with the severity of depressive symptoms (BDI-II and MADRS scores), the ssPERG ratio or the logCS_Weber_. Spearman’s correlation coefficients (*rho*) were computed. *P*-values were calculated by permutations (10,000 replicates). Significance levels in brackets were FDR-adjusted for primary endpoints and not adjusted (^#^) for secondary outcome variables. N indicates the number of complete observations used for analysis

*BDI-II* Beck Depression Inventory II; *FDR* false discovery rate; *logCS*_*Weber*_ logarithmic Weber contrast sensitivity; *MADRS* Montgomery-Åsberg Depression Rating Scale; *N* number of observations; *ns* not significant; *P-ratio* PhNR amplitude at 72 ms/b-wave amplitude; *PhNR* photopic negative response; *W-ratio* (b-wave amplitude − PhNR amplitude at min.)/(b-wave amplitude − a-wave amplitude); * statistically significant difference; ^#^ not adjusted

For the MDD group alone the correlation between the severity of depressive symptoms based on the MADRS scores and the contrast sensitivity was not significant (*rho* = ‒ 0.35; *p* = 0.074). Likewise, no correlation between the MADRS scores and the ssPERG ratio (*rho* = 0.14; *p* = 0.462) was evident in the patient group (Table 3).

#### Contrast sensitivity and ssPERG ratio

Despite both the contrast sensitivity (logCS_Weber_) and the ssPERG ratio being found to be reduced in the MDD group, no correlation between both measures was observed (*rho* = 0.07; *p* = 0.548; Fig. 2C).

#### Retinal ganglion cell responses (ssPERG ratio and the PhNR)

We additionally analyzed the relationship between the ssPERG ratio and the different PhNR measures (Table 3).

A moderate correlation was found between the ssPERG ratio and the PhNR amplitude at min., which had to be considered insignificant after FDR-adjustment of the significance level (*rho* = 0.27; *p* = 0.023). No correlation was observed between the ssPERG ratio and the corresponding W-ratio (*rho* = 0.11; *p* = 0.345).

The PhNR at 72 ms, however, showed a significant moderate correlation with the ssPERG ratio (*rho* = 0.36; *p* = 0.002; Fig. 2D), while the corresponding P-ratio did not show a relation with the ssPERG ratio (*rho* = 0.23; *p* = 0.051).

#### A-wave amplitude of the fERG

Because the a-wave amplitude, as a secondary outcome, was reduced in MDD patients, we further examined the a-wave amplitude for correlations with the BDI-II (MDD and HC) or MADRS (MDD only) scores, the ssPERG ratio, or the logCS_Weber_ (Table 3). This revealed no significant correlation between the a-wave amplitude and the severity of depressive symptoms based on the BDI-II (*rho* =  − 0.23; *p* = 0.063) or the MADRS scores (*rho* = ‒ 0.14; *p* = 0.494), the contrast sensitivity (logCS_Weber_) (*rho* = 0.08; *p* = 0.533), or with the ssPERG ratio (*rho* = 0.07; *p* = 0.589) (Table 3).

#### Antidepressant medication

Due to the small sample size of unmedicated MDD patients (30%), we refrain from inferential and performed descriptive statistics for the subsamples of MDD patients (Supplementary Fig. 1, Supplementary Table 1).

While contrast sensitivity was more reduced for unmedicated (− 0.3; compared to HC) than for medicated MDD patients (− 0.1; compared to HC), the ssPERG ratio was higher for unmedicated (+ 6.7%; compared to HC) than for medicated MDD patients (− 15%; compared to HC). The a-wave amplitude of the fERG was similarly affected for both subgroups of MDD patients (unmedicated MDD vs. HC: − 13.1%; medicated MDD vs. HC: − 14.8%) (Supplementary Fig. 1; Supplementary Table 1).

## Discussion

We examined 30 patients with MDD compared to 42 HC using the Landolt-C contrast test from the FrACT [9] to evaluate the contrast sensitivity (logCS_Weber_) as well as the ssPERG ratio and the PhNR of the fERG to assess retinal ganglion cell response in both groups. The contrast sensitivity and the ssPERG ratio (check size 0.8°/19°) were found to be significantly reduced in MDD compared to HC (by 0.18 for the logCS_Weber_ and by 11% for the ssPERG ratio). The PhNR of the fERG did not differ between groups, while the reduction in the fERG a-wave amplitude in MDD amounted to 15%.

Our finding of a diminished contrast sensitivity in a larger cohort of MDD patients aligns with the results of previous studies [5, 8], which also reported reduced visual contrast discrimination performance and contrast sensitivity in MDD. Accordingly, Chen et al. [57] showed attenuated contrast discrimination sensitivity in patients with subthreshold depression. The observed reduced contrast sensitivity could reflect an altered dopaminergic neurotransmission involved in the pathophysiology of MDD [5, 58].

We further replicated our previous finding of a reduced ssPERG ratio in patients with MDD [24] in an independent larger cohort supporting the hypothesis that the ssPERG ratio serves as a highly valuable measure on the path towards a potential biomarker for MDD. Nevertheless, it should be noted, that compared to our previous study [24], the raw ssPERG amplitudes were not significantly different between MDD and HC, demonstrating the importance of amplitude “normalization” via calculating the ssPERG ratio, thereby reducing interindividual variability.

Compared to our previous study [24], we increased the Michelson checkerboard contrast from 80 to about ≈100%. It might be conceivable that this strong contrast has led to a “ceiling effect” in the retinal ganglion cell responses in MDD and that alterations in the visual processing in depressive patients are likely to occur at perceptual boundaries rather than under optimal stimulation conditions.

Together with the finding of a reduced contrast sensitivity in MDD, we hypothesize that lower Michelson checkerboard contrasts might be more appropriate for ssPERG stimulation in MDD. For instance, Bubl et al. [22] used five different checkerboard contrasts in the range of 3.2% and 80% to calculate the ssPERG contrast gain, corresponding to the increase in ssPERG amplitude with increasing stimulus contrast. They reported a diminished ssPERG contrast gain in patients with acute depressive episodes [22] and a normalization of the ssPERG contrast gain following remission of depressive symptoms [23].

Those results are in line with our present findings of an altered ssPERG ratio in MDD supporting the hypothesis of abnormal retinal ganglion cell responses during depressive episodes. However, Fam et al. [8] only reported a reduced contrast sensitivity in MDD patients but did not find changes in the ssPERG contrast gain in MDD.

Nevertheless, the reduction in the raw ssPERG amplitudes of our MDD patients were more pronounced for the finer compared to the coarser checkerboard pattern, a finding which resembles observations in patients with early glaucoma [25, 26]. But retinal ganglion cell response in MDD might not be abnormal per se since no alterations were found in the PhNR of the fERG. In patients with glaucoma, for instance, both, the ssPERG ratio and the PhNR are similarly affected [48]. In synopsis with the finding of a reduced contrast sensitivity, it would also be conceivable that other mechanisms, like the retinal contrast gain control [59, 60] contribute to visual anomalies in MDD. However, our results show that changes in the retinal ganglion cell response in MDD might be more subtle and determined by the stimulus characteristics than expected.

To facilitate the implementation of ERG measurements in patients with mental disorders, like depression, we applied a handheld ERG device for fERG recordings alongside with PERG examinations. Flash ERGs with the RETeval® would not only shorten the measurement time but also enhance tolerability by using skin instead of corneal electrodes. Moreover, using flashes instead of pattern stimuli would be advantageous, since good visual acuity and proper fixation are less crucial, making the measurement and data quality less dependent on the patient’s cooperation.

While in the fERG no PhNR differences between MDD and HC were detected, we found a significant reduction in the a-wave amplitude in MDD compared to HC, which, however, was only a secondary outcome variable and significance levels did not include FDR-adjustment. Future studies are thus required to confirm the present a-wave results and therewith to further test handheld ERG devices like the RETeval® tool or other promising approaches such as smartphone-based ERG systems [61] as an alternative technique for detecting potential biomarkers for depression or monitor therapy response.

The more laborious PERG method seems to be superior to the fERG using the RETeval® device for the evaluation of retinal ganglion cell responses in MDD. Especially by reducing interindividual variability via calculating an amplitude ratio, the PERG seems to be advantageous. However, within the framework of investigating an objective biomarker, there is a necessity to combine psychophysical and electrophysiological evaluations assessing various stimulus parameters for PERG recordings in order to enhance the distinctions between HCs and patients with a MDD. For example, investigating lower Michelson contrasts for checkerboard stimuli, exploring different stimulation frequencies, or implementing multifocal PERG recordings may facilitate the potential detection of region-specific retinal abnormalities associated with MDD.

### Limitations

Not only untreated patients were examined but also patients who had been on medication with an SSRI, SNRI, or mirtazapine for up to 14 days. However, it was clinically verified by an experienced specialist in psychiatry that a severe depressive episode persisted, and remission had not yet occurred. Based on previous fERG and PERG studies [62] and our subsample analysis of medicated (N = 21) and unmedicated (N = 9) MDD patients, we cannot exclude effects of antidepressive medication on the ssPERG ratio. However, it has to be considered that the median age of our unmedicated (22 years) and medicated (34 years) MDD patients differs about 12 years. The reduction in PERG amplitudes with increasing age is a well-known phenomenon [63] which might possibly be stimulus size specific.

Since the fERG lacks specificity for assessing central retinal function [20], additional techniques such as multifocal flash (mfERG) or PERG testing are necessary to determine if retinal changes in MDD exhibit regional specificity. Measurements were not synchronized with a consistent time of day, precluding the exclusion of circadian-dependent dopaminergic influences [64].

Furthermore, it must be mentioned that impaired concentration and attention, which are important symptoms of MDD patients, may potentially have had an impact on the results, especially regarding the observed reduced contrast sensitivity.

### Summary

We examined 30 patients with a MDD and 42 age- and gender-matched HCs using an optotype-based contrast test, the PERG, and the fERG with the RETeval® device. Patients with MDD exhibited a reduced contrast sensitivity and PERG ratio compared to HC. Moreover, we detected a reduced fERG a-wave amplitude in MDD compared to HC with the handheld RETeval® device. The RETeval® has multiple practical advantages compared to the PERG concerning the measurement process, making it a perfect tool for clinical diagnosis and therapy monitoring. However, before it can substitute the classical PERG confirmatory studies need to be executed.

## Supplementary Information

Below is the link to the electronic supplementary material.

Supplementary file1 (DOCX 142 KB)

## Data Availability

Demographic, psychometric and ERG data as well as the R code for statistical analysis, is available from the corresponding author and EF on request.
