# Supplementary material for: Reduced contrast sensitivity, pattern electroretinogram ratio, and diminished a-wave amplitude in patients with major depressive disorder
Source: Eur Arch Psychiatry Clin Neurosci. 2024 May 28;275(4):1151–63. doi: 10.1007/s00406-024-01826-8 (PMC12149264; doi:10.1007/s00406-024-01826-8)
Supplement: Supplementary file 1 — Supplementary file1 (DOCX 142 KB) [file 406_2024_1826_MOESM1_ESM.docx]

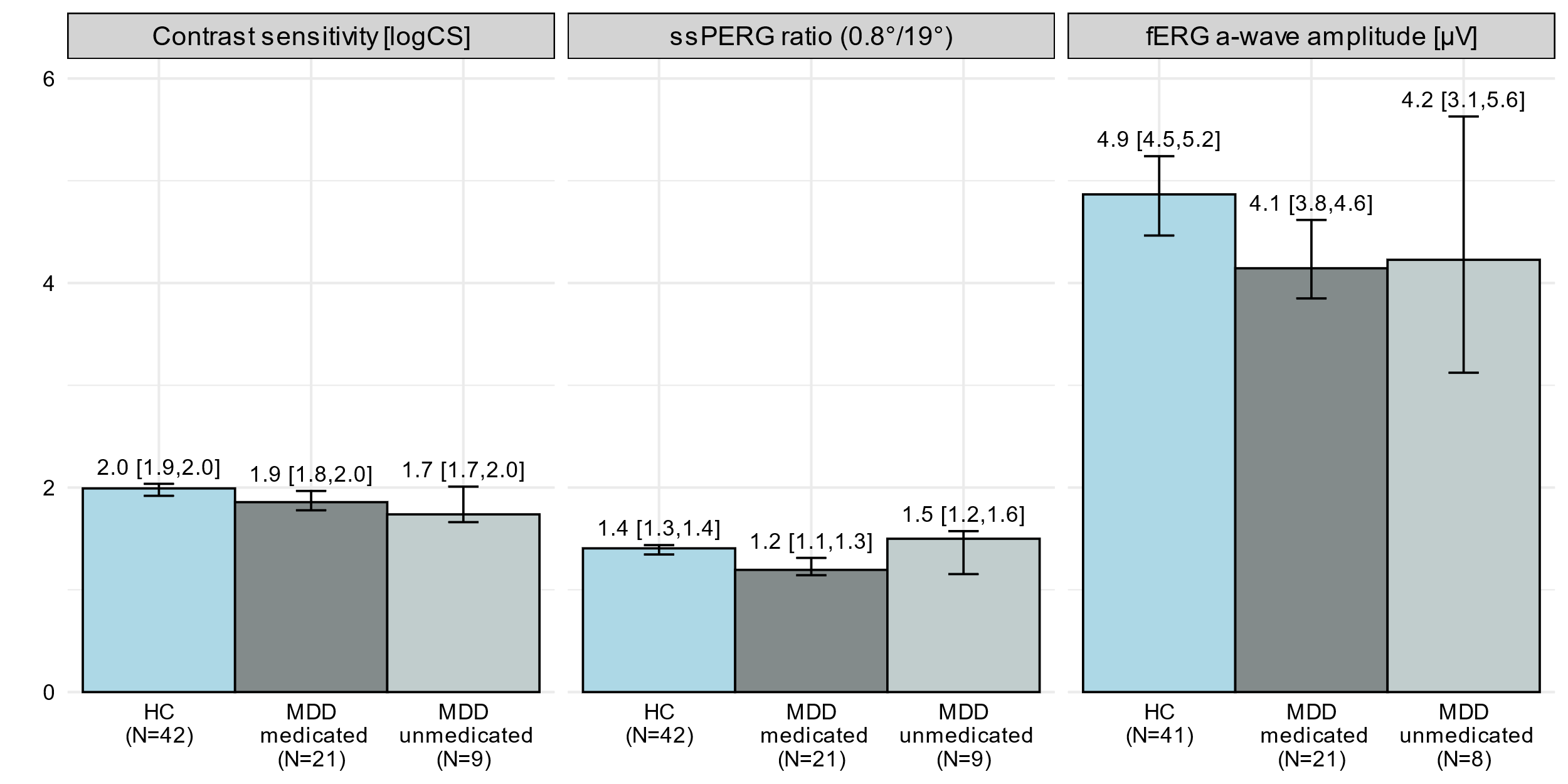


**Supplementary Figure 1:** Contrast sensitivity as logCS_Weber_, ssPERG-ratio and the fERG a-wave amplitude in µV for medicated, unmedicated MDD patients and HCs. Bar heights indicate medians, error bars the bootstrapped (10,000 replicates) 95% confidence intervals (CI). Additionally, medians and CIs (in brackets) are annotated in the bar chart.

*CI* 95% confidence interval; *fERG* flash Electroretinogram; *HC* healthy controls; *logCS_Weber_* logarithmic Weber contrast sensitivity; *MDD* patients with major depressive disorder; *N* number of observations; *ssPERG* steady-state pattern electroretinogram

**Supplementary Table 1:** Descriptive statistics for medicated and unmedicated MDD patients and HCs. The median, the bootstrapped (10,000 replicates) 95% confidence intervals (CI) and the proportional differences in medians between the MDD groups and the HC group (“vs. HC”) are depicted for the ssPERG ratio, the contrast sensitivity, and the a-wave amplitude from the fERG. Additionally, a selection of demographic parameters for the MDD subgroups and the HC group are summarized (counts and proportion in % for factorial, median and 1^st^ and 3^rd^ quartiles for numerical data).

*BDI-II* Beck Depression Inventory II; *CI* 95% confidence interval; *F32.2* severe depressive episode without psychotic symptoms (ICD-10); *F33.2* recurrent depressive disorder, current episode severe without psychotic symptoms (ICD-10); *fERG* flash Electroretinogram; *HC* healthy controls; *ICD-10* International Statistical Classification of Diseases and Related Health Problems version 10; *logCS_Weber_* logarithmic Weber contrast sensitivity; *MDD* patients with major depressive disorder; *MADRS* Montgomery-Åsberg Depression Rating Scale; *N* number of observations; *ssPERG* steady-state pattern Electroretinogram; ^+^ 1 missing data set.

| **Test** | **HC  (N=42)** | **MDD medicated  (N=21)** | | **MDD unmedicated  (N=9)** | |
| --- | --- | --- | --- | --- | --- |
|  | median [CI] | median [95% CI]; | vs. HC | median [95% CI]; | vs. HC |
| Contrast sensitivity [logCS_Weber_] | 2.0 [1.9, 2.0] | 1.9 [1.8, 2.0]; | −0.1 | 1.7 [1.7, 2.0]; | −0.3 |
| ssPERG ratio (0.8°/19°) | 1.4 [1.3, 1.4] | 1.2 [1.1, 1.3]; | −15% | 1.5 [1.2, 1.6]; | +6.7% |
| fERG a-wave amplitude [µV] | 4.9 [4.5, 5.2]^+^ | 4.1 [3.8, 4.6]; | −14.8% | 4.2 [3.1, 5.6]^+^; | −13.1% |
| **Demographical parameter** |  | | | | |
| Sex: female | 29/42 (69%) | 14/21 (67%) | | 7/9 (78%) | |
| Sex: male | 13/42 (31%) | 7/21 (33%) | | 2/9 (22%) | |
| Age in years | 30 (23,35) | 34 (26, 39) | | 22 (20, 29) | |
| ICD-10 Diagnosis: F32.2 | − | 11/21 (52%) | | 5/9 (56%) | |
| ICD-10 Diagnosis: F33.2 | − | 10/21 (48%) | | 4/9 (44%) | |
| Medication duration [days] | − | 7 (4, 11) | | − | |
| BDI-II | 2 (0,4) | 26 (21, 36)^+^ | | 31 (25, 40) | |
| MADRS | − | 36 (35, 38)^+^ | | 40 (36, 40) | |
